# Stent graft coverage of dual-stent strategy in the management of abdominal aortic aneurysms

**DOI:** 10.1038/s41598-018-34354-2

**Published:** 2018-11-05

**Authors:** Yuan Ding, Li Zhongyou, Jiang Wentao, Zhang Yinci, Wang Zhenze, Chen Yu

**Affiliations:** 1 Department of Vascular Surgery of West China Hospital, Sichuan University, Chengdu, 610065 China; 2 Department of Applied Mechanics, Sichuan University, Chengdu, 610065 China; 3 School of Computer science, Sichuan University of Science and Engineering, Zigong, 643000 China; 4 Key Laboratory of Rehabilitation Technical Aids Technology and System of the Ministry of Civil Affairs & Beijing Key Laboratory of Rehabilitation Technical Aids for Old-Age Disability, National Research Center for Rehabilitation Technical Aids, Beijing, 100176 China

## Abstract

Treating an abdominal aortic aneurysm (AAA) with a stent graft (SG) and a multilayer stent (MS) is a key technology in isolating flow fields. Clinically, dual stents (an SG in the proximal and an MS in the distal of AAA) are used for treatment of AAA, but only a few studies have examined the relationship between SG coverage and treatment effects. Through numerical simulation of the hemodynamics after SG and MS implantation, the SG coverage and position were simulated at 0% (0 mm), 25% (13.75 mm), 50% (27.5 mm), and 75% (41.25 mm). With increasing SG coverage, the pressure on the aneurysm sac wall and the flow of branch vessels gradually decreased, and the lower wall shear stress (WSS) gradually increased. The changes in pressure, lower WSS, and the mass flow rate of the branch vessels did not change significantly. The coverage of the SG has a nonsignificant effect on hemodynamics in the treatment of AAA; the implantation position need not be very precise. This research can provide theoretic support for clinicians’ decision-making.

## Introduction

An abdominal aortic aneurysm (AAA) is a life-threatening condition^1^. If they are not treated, AAAs grow and eventually rupture; in such cases, a mortality rate of 80–90% has been reported^2^. Endovascular aneurysm repair (EVAR), which is characterized by blood isolation, has developed rapidly to become the main method of infrarenal AAA treatment since it was first introduced by Parodi^3^ in 1991. However, for some aneurysms that involve significant branches, such as pararenal aneurysm, EVAR is not applicable. The most obvious problem is maintaining the patency of a branching artery; a stent graft (SG) can separate the branch as it isolates the aneurysm, leading to paraplegia and complications such as ischemia of the kidney and other internal organs^4^. The chimney technique^5–8^, defined as EVAR with branching stents, can solve this problem completely because it not only can isolate the aneurysm from circulation but also preserve branch patency. However, the chimney stent usually needs to be custom-made, which is bad for high-risk patients because a lot of time is wasted in the process. In addition, the chimney technique is difficult to complete in clinical operation; hence, new approaches for these aneurysms are still needed.

Multilayer stents (MSs) are three-dimensional self-expandable stents that are fabricated via the cross-linking of metal cobalt alloy wires and were originally used to treat visceral aneurysms. In contrast to the chimney technique, MS is more convenient as doctors can effectively isolate an aneurysm and ensure the blood supply of the branch vessels. Thus, MS is gradually being introduced for the treatment of complicated thoraco-abdominal aortic aneurysms (TAAAs) involving important branches. MS cases were initially reported in France in 2008; a 78-year-old patient with complications such as hypertension occasionally found a large saccular aneurysm in the right renal artery. An MS covered the aneurysm’s neck and postoperative blood flow in the aneurysm sac was significantly reduced, blood flow in the renal artery branch was normal, and the patient’s blood pressure returned to normal. In 2011, an MS was first used to treat Type-B aortic dissection (AD) and was especially effective because of its branching characteristics. MS overcomes the two major defects of SG and brings a new concept to the treatment of AAA involving the branch artery.

Recently, the dual-stent strategy has become a new treatment for AAA and is being used in clinical practice at the West China Hospital of Sichuan University. This strategy combines the advantages of SG and MS for better isolation of AAA. However, the effect of treatment, especially of the SG coverage in the aneurysm sac, has yet to be studied. This study focused on SG coverage in conjunction with an MS. Through a numerical simulation of pressure, wall shear stress (WSS) on the wall of the aneurysm sac, and the impact of blood supply to the branches, this study provides theoretical support for the further application of the dual-stent strategy.

## Models and Methods

**Figure 1:**
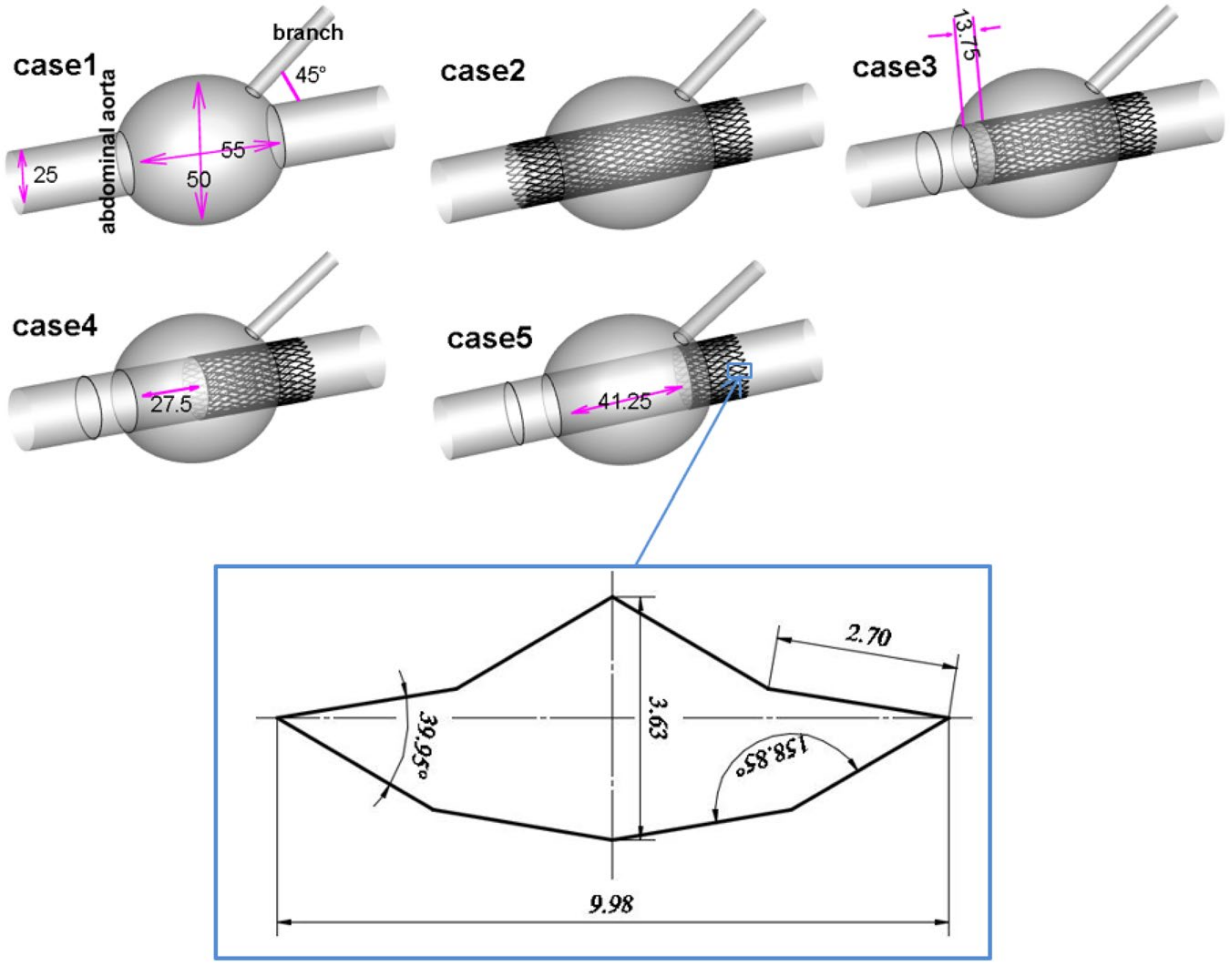
Models. Case1: aneurysm without stent. Case 2: aneurysm with two multilayer stents. Case 3, case 4, and case 5 were aneurysms with a combination of SG and MS, and the coverage rates of SG were 25%, 50%, and 75% respectively. The configuration of the stent element is shown in the box; it was 0.2 mm thick and had double layers (Sinus-XL stent, Optimed, Germany, mesh porosity = 72.2%).

All ideal models were established using SolidWorks software (version 16.0) for qualitative research because of individual differences, as shown in Fig. 1. The diameter of the abdominal aorta was 25 mm, and a branch angled 45° with the aorta was selected, such as the renal artery, with a diameter of 6 mm^9,10^. Stents were implanted in the aneurysm—the SG in front, followed by an MS. The saccular aneurysm was ellipsoid: length of 55 mm and width of 50 mm^10^. The four different SG coverage rates and positions modeled were 0% (0 mm), 25% (13.75 mm), 50% (27.5 mm), and 75% (41.25 mm), respectively, and were marked as case1 (No stent), case2 (0% SG coverage, 100% MS coverage), case3 (25% SG coverage, 75% MS coverage), case4 (50% SG coverage, 50% MS coverage), and case5 (75% SG coverage, 25% MS coverage). After determining the relative positions of the blood vessel and stent, the vessel and stent models were subjected to a Boolean subtraction operation. The stent model was subtracted from the aneurysm model to obtain the vessel model with the stent. In addition, as intraluminal thrombus can impact the simulation, but complex thrombus is significantly different in different people, the existing thrombus inside the capsule was ignored, and an ideal geometry model was designed for computation.

A tetrahedral mesh generated by ICEM (version 16.0) was chosen for the simulation because of its sophisticated geometry, and the command “grid adaption” in the solver Fluent (version 16.0) was used to check for independence of grids to eliminate the sensitivity of the mesh. The final numbers of grid elements were 24,2406, 444,2218, 512,3262, 54,7234, and 566,3312 for case1 to case5, respectively.

To accurately analyze the WSS distribution on the wall of the aneurysm sac and the WSS gradient, a mesh for the fluid boundary layer close to the aneurysm wall is required. For ease of comparison, numerical simulation of the vascular model was also performed with no stent. ANSYS FLUENT (version 16.0) was used for numerical calculation. The equation uses three-dimensional incompressible Navier–Stokes and continuity equations:

1 $$\rho (\overrightarrow{{\rm{u}}}\cdot \nabla )\overrightarrow{{\rm{u}}}+\nabla {\rm{p}}-\mu {\rm{\Delta }}\overrightarrow{{\rm{u}}}=0$$

2 $$\nabla \cdot \overrightarrow{{\rm{u}}}=0$$

Where P and $\overrightarrow{u}$ represent pressure and velocity vectors, respectively. *μ* is the dynamical viscosity of blood, and *∇* and *Δ* represent, respectively, the Hamiltonian and Laplace operators. The inlet parameter uses the fully developed speed boundary conditions given by the User Defined Function (UDF) with an average velocity of 0.12 m/s^11^ (Reynolds number = 900, laminar flow), and the outlet of the aorta and branch was given 11500 Pa and 11000 Pa, respectively^12^. The blood parameters were set to a viscosity of 0.035 Pa·s and a density of 1050 kg/m^3^ ^13^. The blood was a uniform incompressible Newtonian viscous fluid^14^. The walls of the blood vessel and stent were set to a non-slip condition.

## Results

**Figure 2:**
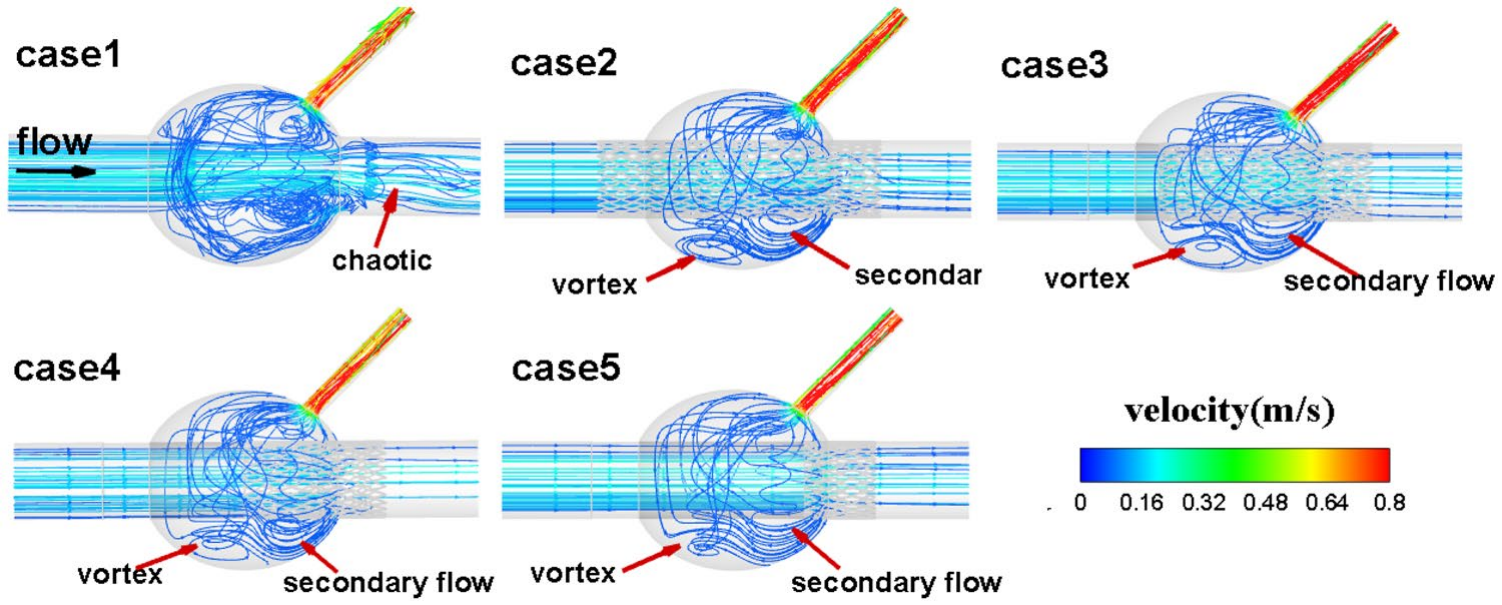
Streamlines in the cases 1–5 models.

The streamlines in the vascular vessels and aneurysm sac of the five models are shown in Fig. 2. The vortex of blood flow is obvious in the aneurysm sac without stent implantation; this vortex is reduced after stent implantation. The streamlines downstream of the aneurysm sac are disordered with no stent. The streamlines in the outlet blood vessels became regular after stent implantation, signifying that the usage of a large porous stent led to non-uniform distribution on the wall of the sac. As SG coverage increased, the vortex in the aneurysm sac was reduced significantly. In addition, the streamlines indicate that the maximum blood flow velocity occurs at the branch of the model blood vessel, ensuring its blood supply.

A significant eddy appears in the anterior part of the aneurysm sac after stent implantation (red arrow in Fig. 2), although there is a strong eddy in the back of the aneurysm sac without stent implantation.

**Figure 3:**
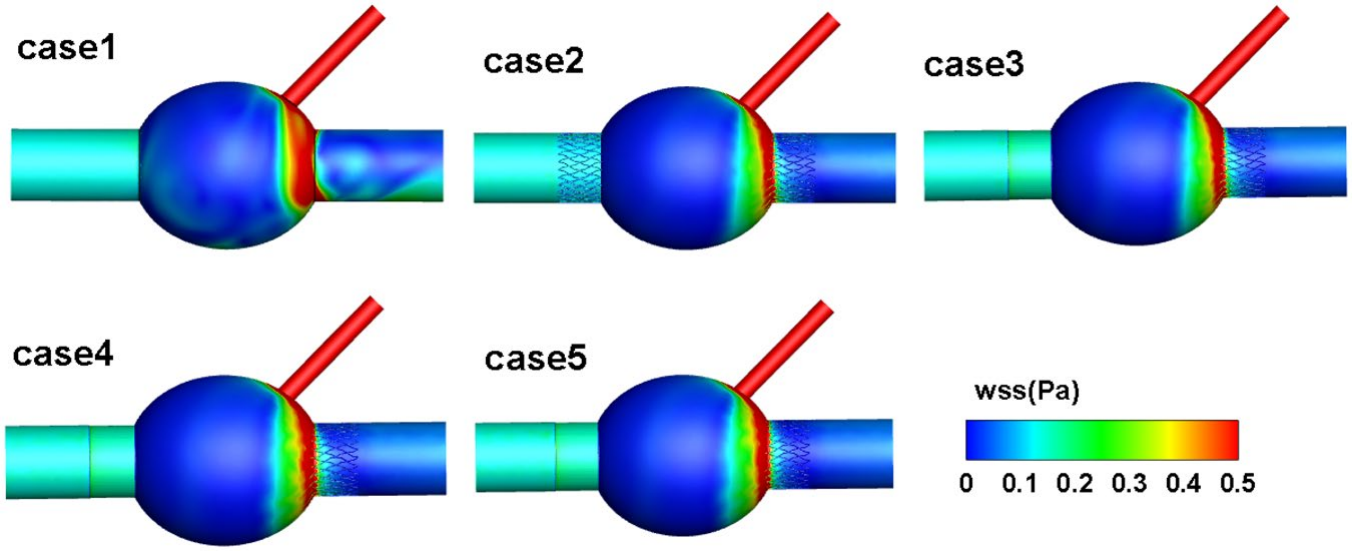
Distributions of WSS in the cases 1–5 models.

The WSS distributions of the five AAA models are shown in Fig. 3. There was a higher WSS value downstream of the aneurysm sac in all five. After stent implantation, the WSS values downstream of the aneurysm decreased. However, the differences in the values of WSS between cases 2 to 5 are not obvious. The WSS distribution in downstream blood vessels without stents is not uniformly influenced by blood flow, and after stent implantation, it becomes very uniform. We calculated five cases with a rigid wall, and the calculated WSS values were relatively small.

**Figure 4:**
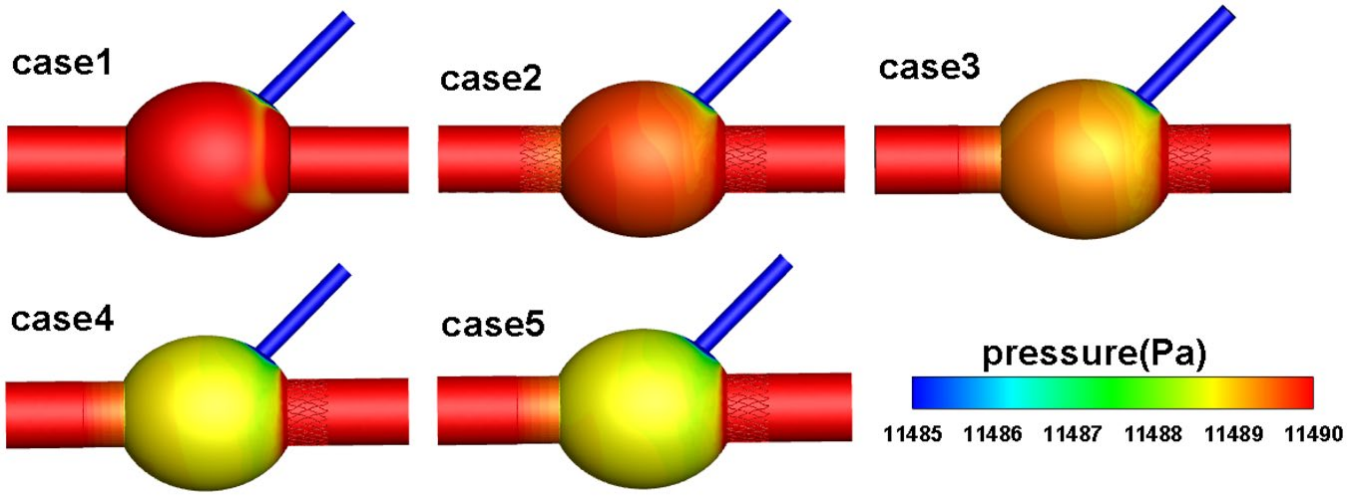
Pressure contour in the cases 1–5 models.

The pressure distributions of the five AAA models are shown in Fig. 4. After implanting the stents, the pressure on the aneurysm sac wall decreased slightly; the pressures on the blood vessel walls and branch blood vessels remained unchanged. The pressure distributions in case 2 are not particularly uniform. However, as SG coverage increases, the pressure on the aneurysm wall gradually became uniform, reducing the risk of aneurysm rupture^15^.

**Figure 5:**
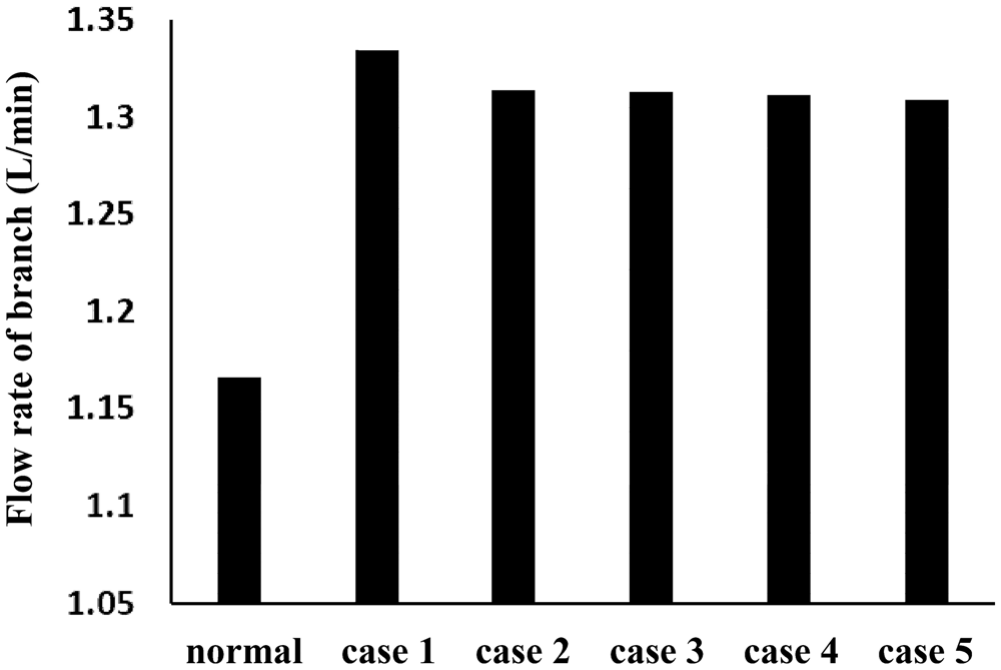
Comparisons of mass flow rates in branch vessels, normal case: without aneurysm.

The branch blood vessels’ mass flow rates are shown in Fig. 5; the flow rates decreased slightly after stent implantation, but the SG position had an insignificant impact on branch blood supply. Compared to branch blood vessel flow without an aneurysm (the “normal” column in Fig. 5), the aneurysm’s formation actually increased the blood supply of the branch vessel, indicating that the aneurysm became a more stable reservoir for blood supply. Even after dual-stent implantation, the flow to the branch vessels is higher than normal.

## Discussions

The efficacy of interventional treatment of AAA is affected by many factors, including shape, porosity, placement, scalability, and hemodynamics parameters. Therefore, various stent strategies should be considered.

For pararenal aneurysm that involves important branches, EVAR may not be applicable, because the branch is blocked while the aneurysm is being isolated, which results in damage to the target organ, such as the kidney. Fortunately, the chimney technique has resulted in a significant breakthrough for treating such aneurysms, but it still has a few disadvantages. For example, the chimney technique usually requires considerable customization time, such as for diameter and angle of branch vessel, because of individual differences. Therefore, it is not suitable for high-risk patients. In clinics, such operations are very difficult for doctors, especially with cases in which several branches are involved. The most complex chimney stent is the octopus, which contains several branch stents. Therefore, finding a more convenient strategy for treating these complicated aneurysms is essential. Recently, interest in MS has increased significantly as a result of its success in clinical application. However, as previous numerical simulations found that two-layer stents had little impact on the pressure on the aneurysm wall^16^, combinations of SG and MS were presented to obtain better isolation, but the coverage of SG still confuses doctors during the intraoperative period.

Another revolution in endovascular repair is fenestrated endovascular aortic repair (F-EVAR), which has realized intra-cavity reconstruction of aortic blood vessels, enabling aneurysms with a short neck (that cannot be treated by conventional techniques) and juxtarenal AAAs (J-AAAs) to receive minimally invasive endovascular therapy. In 2006, O’Neill *et al*. reported J-AAA treatment of 119 F-EVAR-treated patients from the Cleveland Surgery Center in the United States from 2001 to 2005^11^. However, F-EVAR technology is technically demanding, difficult to popularize, and has complications.

Blood flow under the influence of the MS mesh undergo an important change: blood seeped through the stents, the vortex within the sac slowed significantly, the direction of blood flow reversed and gradually returned to laminar flow, and active blood flow gradually undergoes thrombosis such that the aneurysm sac is isolated, enabling the application of blood flow changes to treat aneurysms. The porosity of the MS is an important parameter; the layers of the MS are often used to improve the effect of stents in treating aneurysms. Clinically, 2–4 layers are often used in the MS to separate aneurysms and the effect of the SG, and to maintain the blood supply to the branch vessel. However, lower porosity can degrade the biocompatibility between the vascular wall and the stent and degrade the elasticity of the MS, which is not conducive to the MS’s deformation and the treatment of more complicated aneurysms. Experimental methods comparing five commercial multilayer flow regulators found that stenting reduced aortic wall compliance. Comparing the simulation and particle image velocimetry (PIV) experimental results^17^, pressure and WSS were found to decrease with increasing grid density.

Hemodynamics analysis is helpful in providing theoretical support for doctors, but studies have shown that the coverage of SG has little impact on blood flow, the pressure on the sac decreases slightly, although it became more uniform so as to decrease the risk of stress concentration. In addition, the distribution of WSS on the aneurysm wall also varied slightly although the coverage of SG is different. Thus, the confusion of doctors can be eliminated and an optimal strategy needs to be explored by referring to this simulation. For example, most of the blood flows into the branch through the downstream of the aneurysm, regardless of how much coverage SG provides. Hence, it can be concluded that the SG has no impact on the hemodynamics, especially on pressure. Consequently, positioning the SG in the downstream of the aneurysm may be a better strategy to block the blood, which can provide a new direction for future work.

Finally, a two-layer stent was adopted in the simulation, and the results show that a denser stent is optimal for better isolation and preservation of branch patency at the same time. Interestingly, the flux of the branch is still greater than those of the normal case regardless of whether there are stents or not, because the resistance decreased owing to expansion of the artery (aneurysm).

### Limitation

There are few actual reports in clinical practice, it needs to discuss its feasibility and safety from the ideal aspect, and thus the results presented in this paper are based on ideal models, which differ from the actual situation. Further, this study had several limitations: (1) The existence of thrombosis in the sac was not taken into consideration because of its various forms, which make it difficult to characterize this feature, and so idealized models were adopted. (2) The blood flow in a human is always pulsating, and the corresponding oscillation is always related to time in hemodynamics analysis (we will include this in future work in which we aim to explore some differences between these cases qualitatively). (3) The simulation is only a small step towards maturity; more clinical data and *in vitro* experiments are needed to verify the security and validity of this technique.

## Conclusions

The hemodynamics varied slightly without regard to the coverage of SG, although the pressure on the aneurysm wall became uniform, which eliminates the confusion of doctors. However, this strategy is not suitable for high-risk patients. It can be inferred that a better strategy may be to position the SG downstream of the aneurysm or a high-porosity MS should be selected for dual stents combination application.
